# ASO Author Reflection: Optimizing Lateral Neck Dissection Extent of PTC by FNA-Tg

**DOI:** 10.1245/s10434-021-10636-4

**Published:** 2021-08-12

**Authors:** Xi Jia, Runyi Tao, Ye Yang, Yuanbo Wang, Yan Liu, Aimin Yang, Rui Gao

**Affiliations:** 1 Department of Nuclear Medicine, The First Affiliated Hospital of Xi’an Jiaotong University, Xi’an, 710061 Shaanxi China; 2 Department of Thoracic surgery, The First Affiliated Hospital of Xi’an Jiaotong University, Xi’an, Shaanxi China

## Past

In the past decades, fine-needle aspiration cytology (FNAC) was the primary option for the diagnosis of suspicious lymph nodes on ultrasound in differentiated thyroid cancers (DTC). However, false-negative (6–18%) and non-diagnostic (up to 20%) results are common, resulting in high rates of misdiagnosis^1^.

To improve the diagnostic performance of FNAC, measurement of thyroglobulin (Tg) concentration in the washout fluid of the needle used in FNAC (FNA-Tg) was introduced by Pacini et al. in 1992^2^. FNA-Tg was proved to have high accuracy for detection of nodal metastases from DTC, especially in patients previously treated by thyroidectomy. However, due to the lack of standardization of the sample preparing and assessing procedures, the cutoff value for diagnosis is still under debate. Moreover, the influence of serum Tg and thyroidectomy status on the performance of FNA-Tg is unclear.

## Present

This study highlighted the efficacy of FNA-Tg for detecting lateral neck lymph node metastasis in preoperative papillary thyroid cancer patients^3^. The superior sensitivity and excellent negative predictive values support its routine use as guidance for lateral neck dissection. Because the level of FNA-Tg in the central compartment was easier to be by affected blood contamination or Tg leakage than that in the lateral compartment^4^^,^^5^, the additional diagnostic value of FNA-Tg is limited in central neck dissection planning. It is of note that, as compared to the absolute value, no diagnostic advantage was found for the difference or ratio between FNA-Tg and serum Tg.

A standardized FNA-Tg sample preparing procedure was introduced in this study^3^, a fixed 5 µL of aspiration was added to 195 µL of Tg-free serum3. This could be a potential standard operating procedure with a fixed volume of aspiration sample and diluent matrix, thereby enabling comparisons among studies. Future multicenter data for a large-scale trial could provide robust evidence of diagnostic accuracy to the standard operating procedure of FNA-Tg.

## Future

FNA-Tg alone could accurately diagnose lateral lymph node metastasis in this study^3^. This might be a convenient and inexpensive choice in the following clinical application. Whereas an algorithm may be useful for future central lymph node diagnosis, including FNA-Tg, FNAC, and intraoperative frozen pathology.
